# A randomized clinical trial of unfractioned heparin for treatment of sepsis (the HETRASE study): design and rationale [NCT00100308]

**DOI:** 10.1186/1745-6215-7-19

**Published:** 2006-05-26

**Authors:** Fabián Jaimes, Gisela De La Rosa, Clara Arango, Fernando Fortich, Carlos Morales, Daniel Aguirre, Pablo Patiño

**Affiliations:** 1 Associate Professor, Department of Internal Medicine and Grupo Académico de Epidemiología Clínica, School of Medicine, Universidad de Antioquia, Medellín, Colombia. Doctoral candidate, Department of Epidemiology, Johns Hopkins Bloomberg School of Public Health, Baltimore, MD, USA; 2 Assistant Professor, Department of Internal Medicine, School of Medicine, Universidad de Antioquia, Medellín, Colombia; 3 Professor, Department of Surgery and Grupo Académico de Epidemiología Clínica, School of Medicine, Universidad de Antioquia, Medellín, Colombia; 4 Associate Researcher, Grupo Académico de Epidemiología Clínica, School of Medicine, Universidad de Antioquia, Medellín, Colombia; 5 Professor, Department of Microbiology and Grupo de Inmunodeficiencias Primarias, School of Medicine, Universidad de Antioquia, Medellín, Colombia

## Abstract

**Introduction:**

Infection promotes coagulation via a large number of molecular and cellular mechanisms, and this procoagulant activity has boosted basic and clinical research using anticoagulant molecules as therapeutic tools in sepsis. Heparin, which is a naturally occurring proteoglycan that acts by reducing thrombin generation and fibrin formation, has not been rigorously tested in a randomized clinical trial.

**Methods:**

Randomized, double-masked, placebo-controlled, single-center clinical trial. Patients are recruited through the emergency room at Hospital Universitario San Vicente de Paul. This is a 650-bed University Hospital in Medellín, Colombia and is a referral center for a region with approximately 3 million habitants. The recruitment process started on July 2005 and will finish on June 2007. Patients aged 18 years or older, males or females, hospitalized with clinically or microbiological confirmed sepsis, have been included. The interventions are unfractioned heparin in low dose continuous infusion (500 units per hour for 7 days) or placebo, additionally to the standard of care for sepsis patients in Colombia.

**Results:**

Our primary aims are to estimate the effects of heparin on hospital length of stay and change from baseline Multiple Organ Dysfunction (MOD) score. Secondary objectives are to estimate the effects of heparin on 28-day all-cause mortality, and to estimate the possible effect modification on 28-day all-cause mortality, in subgroups defined by source and site of infection, and baseline values of APACHE II score, MOD score and D-dimer.

**Conclusion:**

The available literature in animal and human research, and the understanding of the molecular biology regarding inflammation and coagulation, supports a randomized clinical trial for the use of heparin in sepsis. Our study will provide appropriate power to detect differences in valid surrogate outcomes, and it will explore important preliminary data for efficacy regarding the clinical end-point of mortality.

## Background

Sepsis is considered a leading cause of death worldwide with approximately 18 millions cases annually and a mortality rate of almost 30% [1]. In Colombia, the country in which this study will be conducted, there are no national estimates of sepsis incidence. However, our previous investigations have provided some relevant data: in a 650 bed University-based Hospital, bacteremia and/or sepsis were considered a main diagnosis in 7 out of 100 admissions to the emergency room; and blood cultures were requested in 2 out of 10 inpatients at some time during their hospitalization [2,3]. The overall 28-day sepsis mortality rate ranged from 25% to 40%, depending on the growth of microorganisms in blood cultures [2-4].

In the last two decades the accepted standard treatment for sepsis has resulted in only a slight decrease in mortality, and that decrease has been overshadowed by an almost 300% increase in incidence [5]. Unfortunately, the search for efficacious therapeutic approaches has largely failed and only a few of the recent interventions -as early goal directed therapy, activated protein C and low dose steroids- have shown success in improving survival [6,7]. However, these interventions were tested only in patients with severe sepsis and septic shock and although these groups exhibit the highest mortality, they may represent less than 50% of the total affected population [8,9]. Furthermore, these interventions necessitate special devices, tests or drugs that might be unavailable or simply unaffordable in resource-limited settings.

Several investigators have documented the close relationship between infection, inflammation and coagulation in sepsis [10-12]; and although clinically overt disseminated intravascular coagulation (DIC) may occur in only 30%–50% of septic patients, the activation of coagulation cascade is an early and common response to the infectious challenge [13-15]. In turn, most of the molecules involved in the pro-coagulant state that characterizes sepsis are also powerful generators or amplifiers of the inflammatory response [16,17]. The rationale behind anticoagulant treatments is that certain factors -activated protein C (APC), Antithrombin (AT) and Tissue Factor Pathway Inhibitor (TFPI) – are depleted, and the use of recombinant technology may replenish them. In contrast, heparin does not simply replenish what sepsis patients have depleted, it binds to and activates AT. As a consequence of this activation, heparin dramatically reduces thrombin generation and fibrin formation. Rather than artificially providing a component in the regulatory system, heparin takes advantage of the one existing molecule in its natural environment to increase its activity a thousand-fold [18].

Animal and human models have suggested that heparin, in addition to successfully inhibiting the coagulation cascade in sepsis, may also modulate a wide array of responses to infection [19-23]. Furthermore, the three clinical trials for recombinant anticoagulants allowed the use of prophylactic treatment for venous thrombosis with a dose of heparin of up to 10,000 or 15,000 units subcutaneously per day [24-26]. Although heparin was not given based on a random allocation, when those who did receive heparin were compared to those who did not in the placebo arms of the clinical trials, all three studies showed a higher mortality in the subgroups that did not receive heparin. Despite the obvious limitations of post-randomization comparisons, a constant result in three different study populations with variable entry criteria, along with the natural heterogeneity of the illness, strongly fosters the hypothesis that heparin might reduce, beyond its known anticoagulant and antithrombotic properties, the overall mortality for sepsis.

As noted in a recent editorial in JAMA [27], heparin is the most widely available, least expensive, and most frequently used anticoagulant; and despite the common recommendation of continuous infusion of low doses (300–500 units/hour) in the treatment of DIC [13,28], its potential and attractive usefulness as therapy for the treatment of sepsis has not been rigorously tested in a randomized clinical trial. Therefore, we are conducting a clinical trial for testing low dose continuous infusion of unfractioned heparin (500 units/hour for 7 days) as complementary treatment for septic patients.

Our primary aims are to estimate the effects of heparin on hospital length of stay and change from baseline Multiple Organ Dysfunction (MOD) score [29]. Secondary objectives are to estimate the effects of heparin on 28-day all-cause mortality, and to estimate the possible effect modification on 28-day all-cause mortality, in subgroups defined by source and site of infection, and baseline values of APACHE II score, MOD score and D-dimer.

## Materials and methods

### Study design

Randomized, double-masked, placebo-controlled, single-center clinical trial.

### Population

An ideal therapy should be viable for septic patients at the earliest stage in their clinical course once the infectious process is suspected or confirmed, and not only at the point of admission to intensive care as has been the case for virtually all previous sepsis trials. Although the definitions for severe sepsis and septic shock have been relatively easy, the identification of that "less ill" population has been really hard to meet. In fact, currently we are lacking a standard definition for sepsis. We have shown [30] that in the setting of the proposed study, the classical systemic inflammatory response syndrome (SIRS) definition was not inclusive enough to identify the full spectrum of patients, since its sensitivity was less than 70% for a cohort with a median length of stay of 11 days and an overall hospital mortality of 21%. Thus, we are proposing a set of inclusion criteria that may be useful to include the wide spectrum of the syndrome. In addition to fulfilling the operational criteria for inclusion, we are restricting our population to the patients with a clinical picture as severe enough to require inpatient treatment.

Patients will be recruited, between July 2005 and June 2007, through the emergency room at Hospital Universitario San Vicente de Paul. This is a 650-bed University Hospital in Medellín, Colombia and is a referral center for a region with approximately 3 million habitants. Patients aged 18 years or older, males or females, hospitalized with a suspected or confirmed infection, unexplained fever, unexplained altered mental status or unexplained arterial hypotension (systolic blood pressure < 90 mm Hg or a decrease > 40 mm Hg) will be considered potentially eligible for the trial.

### Inclusion criteria (table 1)

**Table 1:** Inclusion criteria – HETRASE study

**Modified CDC definitions for infection**	**General variables**	**Inflammatory variables**
1) Pneumonia	1) Temperature (oral or axillary) > 38°C or < 36°C	1) WBC > 12,000 μL^-1 ^or < 4,000 μL^-1 ^or with > 10% immature forms
2) Bloodstream infection	2) Heart rate > 90 beats/min	2) Plasma C-reactive protein > 5 mg/dL.
3) Clinical sepsis	3) Respiratory rate > 20 breaths/min	
4) Symptomatic urinary tract infection and other infections of urinary tract	4) Altered mental status determined by Glasgow Coma Scale < 15	
5) Intra-abdominal infections	5) Systolic blood pressure < 90 mm Hg or a decrease > 40 mm Hg	
6) Skin infections		
7) Soft tissue infections		
8) Superficial and deep surgical site infections		
9) Joint or bursa infections		

- Patients must have an infection defined by clinical and/or microbiological criteria in accordance with modified CDC definitions for nosocomial infections [31].

- Patients must present with one or more of the general variables *AND *one or more of the inflammatory variables, within 24 hours before admission to the study. These variables should not be attributable to an underlying disease other than infection or due to the effects of concomitant therapy.

### Exclusion criteria (table 2)

**Table 2:** Exclusion criteria – HETRASE study

- Pregnant or breastfeeding.
- Platelet count < 60,000/mm^3^.
- Increased risk for bleeding: a) Any patient who has undergone major surgery, defined as surgery that required general or spinal anesthesia, performed within the 12-hour period immediately preceding admission to the hospital; any postoperative patient who demonstrates evidence of active bleeding; or any patient with planned or anticipated major surgery during the first 12 hours after admission to the hospital. b) History of: severe head trauma that required hospitalization, intracranial surgery, or stroke within 3 months of study entry; or any history of intracerebral arteriovenous malformation, cerebral aneurysm, or central nervous system mass lesion. c) History of congenital bleeding diatheses, such as hemophilia. d) Gastrointestinal bleeding within 6 weeks of study entry that required medical intervention unless definitive surgery has been performed. e) Trauma patients at increased risk of bleeding, for example: flail chest; significant contusion to lung, liver, or spleen; retroperitoneal bleed; pelvic fracture; or compartment syndrome.
- Patients with a known hypercoagulable condition including activated Protein C resistance; a hereditary deficiency of Protein C, Protein S, or antithrombin; presence of anticardiolipin antibody, antiphospholipid syndrome, lupus anticoagulant or homocysteinemia; or patients with a recently documented (within 3 months of study entry) or highly suspected deep venous thrombosis or pulmonary embolism.
- Patients taking or requiring the following medications: a) Therapeutic heparin, defined as UFH dosed to treat an active thrombotic or embolic event within the 12 hours prior to study entry or LMWH used at any dose higher or more frequent than the recommended dose on the product label for prophylaxis within the 12 hours prior to study entry. b) Warfarin, if used within 7 days of study entry. c) Thrombolytic treatment within 3 days of study entry (for example, streptokinase, rtPA, and urokinase). d) Glycoprotein IIb/IIIa antagonists within 7 days of study entry.
- Patients with known esophageal varices, chronic jaundice, cirrhosis, or chronic ascites.
- Presence of an advance directive to withhold life-sustaining treatment
- Patients not expected to survive 28 days given their preexisting, uncorrectable medical condition. This criterion includes patients with, or suspected to have, poorly controlled neoplasms or other end-stage processes, such as end-stage cardiac disease, prior cardiac arrest, end-stage lung disease, or end-stage liver disease.
- Patients with chronic renal failure on either hemodialysis or peritoneal dialysis.
- HIV positive patients with most recent CD4 count < 200/mm^3^.
- Patients who have undergone bone marrow, liver, lung, kidney or pancreas transplantation.
- Inability or unwillingness of patients or legal representative to give written informed consent.

- Pregnant or breastfeeding.

- Platelet count < 60,000/mm^3^.

- Increased risk for bleeding

- Patients with a known hypercoagulable conditions

- Patients taking or requiring anticoagulant medications

- Patients with known esophageal varices, chronic jaundice, cirrhosis, or chronic ascites.

- Presence of an advance directive to withhold life-sustaining treatment

- Patients not expected to survive 28 days given their preexisting, uncorrectable medical condition.

- Patients with chronic renal failure on either hemodialysis or peritoneal dialysis.

- HIV positive patients with most recent CD4 count < 200/mm^3^.

- Patients who have undergone bone marrow, liver, lung, kidney or pancreas transplantation.

- Inability or unwillingness of patients or legal representative to give written informed consent.

### Randomization and following procedures

The treatment assignment ratio is 1:1, fixed throughout the study. The allocation to heparin or placebo is defined by randomly permuted blocks of size 2, 4 and 6 generated by a random number generator (*ralloc *program, Stata co. 8.2, College Station, TX, USA). Complete treatments (four ampoules for each patient, marked with the same sequential number corresponding to the randomization scheme) are available in the emergency room's pharmacy center. The placebo is packed identically to the heparin sodium injection, and has the same color and volume as the heparin. Therefore, procedures for administration (i.e. loading dose and continuous infusion) are identical. Outcome measures are documented by medical research assistants masked to both the subject's intervention group assignment and the activated partial thromboplastin time (aPTT) values corresponding to specific days of the intervention period.

### Methods for dealing with potential adverse events

General treatment for patients is left up to the medical team in charge of each patient, according to clinical practice guidelines for infectious diseases and critical care available at the institution.

Basic considerations for dealing with potential adverse events are:

- Infusion should be suspended indefinitely if there are *new *signs or symptoms of hemorrhage such as bleeding from gums, nosebleeds, extensive bruising, evidence of purplish skin areas or gastrointestinal bleeding.

- Infusion should be suspended indefinitely if there is an otherwise unexplained drop of 50% or greater in platelet count, or an absolute platelet count below 30,000 cells per mm^3 ^any time during the study.

- Infusion should be interrupted 2 hours before any percutaneous procedure or major surgery, and should only be resumed 1 hour after a percutaneous procedure or 12 hours after major surgery. In presence of bleeding complications with any of these procedures, infusion can only be resumed after normal results in coagulation tests, and no less than 24 hours after the procedure

- Infusion should be interrupted indefinitely if aPTT test is prolonged more than 60 seconds at any time during the infusion period.

### Sample size

Length of stay may be considered a time to event end-point, with patient discharge alive as the outcome. Those who die during the hospital stay will be considered as competing risks for this analysis because they are not censored observations. With a median length of stay of 11 days, a type I error of 0.05 and a type II error of 0.2, the estimated number of patients required to detect a relative hazard of being discharged alive of 1.3 is 231 [32]. This number of events may be achieved with a total sample size of 310 patients, assuming an overall hospital mortality of 25%.

MOD score data correspond to the case of longitudinal data with correlated measurements, and as such require special considerations for calculation of power and sample size [33]. The table 3 shows different estimates of power under different assumptions of correlation among repeated observations (ρ) and differences in rate of change (Δ) with a fixed sample size of 310 participants. This sample size also will have an 80% power to detect a 50% relative reduction in the secondary outcome of overall 28-day mortality.

**Table 3:** Power estimates for differences (Δ) in MOD score with a fixed sample size of 155 patients per group and selected values of correlation (ρ)

ρ Δ	3 points	2 points	1 point
0.8	100%	99%	98%
0.5	100%	98%	97%

### Interim monitoring

An independent Data and Safety Monitoring Board (DSMB) comprising three members with expertise in statistics, epidemiology and critical care practice are responsible for the interim monitoring process. The statistician in the data-coordinating center is the only person with access to the blind and the full data and he provides the required information, both overall for the interim quality assurance and specific by treatment group (efficacy and safety) exclusively for the DSMB.

The first interim was conducted when the first 103 study participants completed their 28-day vital status evaluation -approximately 9 months after study recruitment began. The second interim analysis will be conducted when 206 patients have been enrolled, approximately 16 months after the study initiation. Stopping guidelines for efficacy monitoring were determined according to the modified O'Brien-Fleming procedure [34,35]. With this procedure, the values for statistical significance will be 0.0006 and 0.0151 in the first (α_1_) and second (α_2_) interim analyses, respectively, for a final significance (α) of 0.0471.

### Analysis plan

Overall study efficacy will be established using the intention-to-treat principle, in which patients are analyzed in the treatment group to which they are assigned at randomization. Length of stay may be considered a time to event time end-point with discharge as the outcome. The graphical presentation of these survival distributions for heparin and placebo groups will be estimated using the Kaplan-Meier method, and the test statistic for comparison between these two survival distributions will be based on the Log-rank test [32].

MOD score data correspond to the case of longitudinal data with correlated measurements, and as such require special statistical methods to account for the correlation among observations. To analyze this kind of clustered data, it is necessary to model both the regression of *Y *(the outcome measure) on *x *(the intervention) and the within-cluster dependence in a unified manner with the application of a random-effects model for longitudinal data [36].

For secondary outcomes, the comparisons will be presented as absolute and relative differences in 28-day mortality rates with exact 95% confidence intervals. The statistic to test this secondary null hypothesis will be the Chi-square test. Although the expectation is that randomization assists in balancing known and unknown prognostic factors, this assignment process is not a guarantee against the possibility that estimates of treatment effect will be modified by covariate imbalance. Accordingly, the estimated relative risk of mortality for heparin vs. placebo will be adjusted by the most important prognostic factors (age, APACHE II score and MOD score at baseline), using a multiple logistic regression model.

The natural heterogeneity of sepsis supports the importance of exploring the efficacy of the intervention among different subgroups of patients, as defined by characteristics of the illness. As hypothesis-generating analysis, the following interaction terms will be incorporated into the logistic model described before:

- Treatment * Source of infection (pneumonia vs. others)

- Treatment * Site of acquisition (community vs. hospital)

- Treatment * APACHE II score at baseline (continuous variable)

- Treatment * MOD score at baseline (continuous variable)

- Treatment * D-dimer at baseline (continuous variable)

### IRB approvals

The protocol and the informed consent document were reviewed and approved by the ethics committee responsible for oversight the study (Medical Research Center, Universidad de Antioquia -U de A-, Medellín, Colombia), and by the Committee on Human Research at Johns Hopkins Bloomberg School of Public Health (CHR#: H.34.04.04.27.B1)

## Discussion

Sepsis is a leading cause of morbidity and mortality worldwide, and it is reasonable to assume that the magnitude of the problem is accentuated (i.e., higher costs and burden of disease) in developing countries. Thus, considering the problem of resource-limited settings, ideal characteristics of therapies that complement standard care might include the following: efficacious, effective, simple and inexpensive. Therapies should be void of adverse events as much as possible, and should be widely available [37]. Therefore, we will test the hypothesis that unfractioned heparin (low-dose continuous-infusion: 500 units/hour during 7 days) is efficacious as a complementary treatment in patients with signs indicative of sepsis. This dose was selected based on the best risk/benefit ratio. It has the ability to activate AT and reduce fibrin without the potential risks of full anticoagulation. In addition, this amount is equivalent to the dose widely used as prophylactic treatment for patients considered at risk for venous thrombosis (10,000 to 15,000 units subcutaneously per day).

Several animal and preclinical studies have explored the potential benefits of heparin in bacterial infections. Meyer and coworkers hypothesized that heparin may increase cardiac output and oxygenation [20]. Fourteen sheep received continuous infusion of *Escherichia coli *endotoxin over 24 hours. Seven animals received a fixed dose of 5,000 units of heparin every 4 hours immediately after starting the endotoxin infusion and the other seven sheep served as controls. The heparinized animals showed a tri-phasic cardiovascular response, characterized by increase in cardiac index and decrease in systemic vascular resistance in the first two hours, followed by a return to approximately baseline levels at four hours. In the last phase (8 to 24 hours), cardiac index increased and systemic vascular resistance decreased significantly, accompanied by a marked improvement in oxygenation variables (arterial oxygen tension and mixed venous oxygen saturation) compared to the control group.

Schiffer et al compared hirudin, a specific and extremely potent thrombin inhibitor, to heparin in terms of endotoxin-induced DIC and subsequent multiple organ failure [38]. Twenty-two sheep were allocated to one of three treatment groups: a) normal saline; b) continuous infusion with conventional unfractioned bovine lung heparin; and c) continuous infusion with recombinant hirudin. After a 6-hour period of baseline evaluation, *Escherichia coli *endotoxin was administered. Infusion of endotoxin was lethal for all sheep allocated to the control and hirudin groups, whereas four of the seven animals receiving the continuous heparin infusion survived to the end of the study. In the control group, animals died between 6 and 44 hours of the start of endotoxin administration and between 8 and 30 hours in the hirudin group. The three deaths in the heparin group occurred between 48 and 56 hours following the start of endotoxin administration, and the difference in survival rate as compared with the two other treatment groups was statistically significant.

The most noteworthy finding in this study was the inability of hirudin to protect animals from the development of DIC, and to prevent multiple organ failure and subsequent death in a manner similar to that of heparin. The authors offered several possible explanations of these results: the time course of thrombin-antithrombin complex production indicates that hirudin was less efficient than heparin in preventing thrombin generation. Given that hirudin acts directly on free thrombin, it is possible that the amount of thrombin neutralized by hirudin is not enough to slow its generation through the activated factor X, and eventually, other co-factors such as V and VIII. Additionally, the heparin-AT complex reduces the endotoxin-induced generation of plasminogen activator inhibitor type 1 (PAI-1), thus favoring a pro-fibrinolytic effect not observed with hirudin [38]. Although the main action of heparin results from the inactivation of clotting proteases by AT, some inhibitory actions are independent of coagulation. Instead, they seem related to a large-spectrum of nonspecific enzyme inhibition activity [16].

In 1983, Haneberg et al conducted the first controlled clinical trial in humans to evaluate heparin, studying 26 infants and children with severe meningococcal sepsis [39]. Eleven patients received intravenous heparin "as early as possible following admission to hospital" and continuously for two days. Fifteen children received only the standard treatment. There were two deaths in each group and the clinical course of the surviving patients did not exhibit significant improvement. The study did not include data regarding time before admission, clinical severity, comorbidities, co-interventions or other potential important differences between groups.

Pernerstorfer et al [22] studied 30 healthy male volunteers in three groups of 10 subjects. All 30 study subjects received lipopolysaccharide (LPS) 2 ng/kg IV. Ten minutes after LPS infusion, subjects in the heparin group received 80 IU/kg, followed by a continuous infusion at a rate of 18 IU/kg/hour for 6 hours, and subjects allocated to the low molecular weight heparin (LMWH) group received dalteparin 40 IU/kg followed by continuous infusion of 15 IU/kg/hour. Ten subjects served as controls. Activation of coagulation, as a consequence of LPS infusion, caused marked increases in plasma levels of prothrombin fragment F_1+2 _(10-fold) and polymerized soluble fibrin (6-fold) in the placebo group. LPS infusion also increased subjects' D-dimer levels in the same group (5-fold). In contrast, heparin infusion completely abolished F_1+2 _generation and D-dimer production, whereas F_1+2 _and D-dimer levels increased approximately 2-fold in the LMWH group. In accordance with the primary role of tissue factor (TF) on coagulation activation in sepsis, TF-positive monocytes doubled after LPS infusion in the placebo group. Interestingly, heparin completely blocked this LPS-induced increase in TF-positive monocytes.

Working with the same study population described above, Derhaschnig et al reported their findings on early inflammatory responses [23]. Neutrophil and platelet counts decreased by a maximum of 15% between 70 minutes and 112 minutes after LPS infusion and these changes were not modified by any treatment. In contrast, LPS-induced lymphocytopenia was significantly less pronounced in the heparin group. In concordance with this result, the expression of L-selectin, an adhesion molecule with a pivotal role in lymphocyte homing, decreased by 32% in the placebo group after 6 hours but only by 5% and 24% in the heparin and LMWH groups, respectively. Tumor necrosis factor alfa (TNF-α) levels increased > 350-fold in the LMWH and placebo groups, but only 150-fold in the heparin group (*p *= 0.07). C-reactive protein levels (CRP) were 4.5 mg/dL in the placebo group (range 2.8–6.1), 3.9 mg/dL in the LMWH group (3.0–4.8) and 3.0 mg/dL (1.8–4.0) in the heparin group.

Boldt et al conducted a study to assess whether continuous heparinization influenced plasma levels of circulating adhesion molecules in intensive care patients [21]. Twenty-eight trauma patients and 28 patients who had developed sepsis secondary to major abdominal surgery were included in the study. Heparin was administered intravenously (600 units/hour) to 14 patients in each group -trauma and sepsis- approximately 24 hours after arrival in the intensive care unit, with an equal number of subjects serving as controls. The following adhesion molecules were measured daily: soluble endothelial leukocyte adhesion molecule-1 (sELAM-1), vascular cell adhesion molecule-1 (sVCAM-1), intercellular adhesion molecule-1 (ICAM-1) and soluble granule membrane protein 140 (sGMP-140). There were no significant differences between groups. There was no investigation of organ dysfunction or septic shock during the period of study, and with the exception of platelet count and the Pao_2_/Fio_2 _index, no other relevant hematological, biochemical or inflammatory parameters were followed.

In summary, experimental models of human endotoxemia have demonstrated a wide range of ways in which heparin may block the pro-coagulant activity that characterizes sepsis. But the expression of a subsequent inhibition in the inflammatory pathways has not been as clearly illustrated in human models as it has been in animal models. On the other hand, in the clinical setting of sepsis, neither the anticoagulant effect nor the broad anti-inflammatory and/or catalytic properties of heparin have been appropriately evaluated. The measurements of organ dysfunction over time and hospital length of stay may accomplish this goal, as a first approach to determine the potential efficacy of the drug.

## Conclusion

The available literature in animal and human research, and the understanding of the molecular biology regarding inflammation and coagulation, supports a randomized clinical trial for the use of heparin in sepsis. Our study will provide appropriate power to detect differences in valid surrogate outcomes, and it will explore important preliminary data for efficacy regarding the clinical end-point of mortality.

## Key messages

- A pro-coagulant state is the hallmark in the inflammatory response that characterizes sepsis.

- Some biological products with anticoagulant activity have been tested in clinical trials, as complementary treatment for septic patients.

- Several animal and pre-clinical studies have suggested potential benefits for unfractioned heparin in bacterial infections.

- A randomized clinical trial of heparin in sepsis is on course, and it will provide important preliminary data for efficacy and safety.

## Competing interests

The author(s) declare that they have no competing interests.

## Authors' contributions

FJ conceived the study, participated in its design and drafted the manuscript. GDLR, CA, FF, CM, DA, and PP participated in the design. All authors read and approved the final manuscript.
